# Role of Inflammatory/Immune Response and Cytokine Polymorphisms in the Severity of Chronic Hepatitis C (CHC) before and after Direct Acting Antiviral (DAAs) Treatment

**DOI:** 10.3390/ijms24021380

**Published:** 2023-01-10

**Authors:** Joana Ferreira, Mariana Oliveira, Manuel Bicho, Fátima Serejo

**Affiliations:** 1 Institute for Scientific Research Bento Rocha Cabral, Calçada Bento da Rocha Cabral 14, 1250-012 Lisboa, Portugal; 2 ISAMB, Genetics Laboratory, Lisbon Medical School, University of Lisbon, Av. Professor Egas Moniz, 1649-028 Lisboa, Portugal; 3 Gastroenterology and Hepatology Department, Hospital de Santa Maria, Av. Professor Egas Moniz, 1649-028 Lisboa, Portugal

**Keywords:** chronic hepatitis C, DAA treatment, liver fibrosis, anti- and pro-inflammatory cytokines, genetic polymorphisms

## Abstract

Host regulatory immune response is involved in the hepatic inflammatory process caused by the hepatitis C virus (HCV). We aimed to determine if HCV clearance with direct-acting antivirals (DAAs) changes the hepatic fibrosis stage, biochemical parameters of liver injury, and inflammatory/immune responses. Sample: 329 chronic hepatitis C (CHC) patients, 134 of them treated with DAAs. Liver fibrosis was evaluated by transient elastography (FibroScan), biochemical and cellular parameters were determined by standard methods, cytokine concentration by enzyme-linked immunoabsorbent assay (ELISA), and genetic polymorphisms by polymerase chain reaction—restriction fragment length polymorphism (PCR-RFLP) or endpoint genotyping. Before DAA treatment, severe fibrosis or cirrhosis (F3/4) was associated with higher values of tumor necrosis factor-alpha (TNF-α) and genotypes transforming growth factor-beta-509 C/T_CC (TGF-β-509 C/T_CC), interleukine-10-1082 T/C_CC (IL-10-1082 T/C_CC), and IL-10-592 G/T_GT. After DAA treatment, fewer F3/4 patients and lower values of TNF-α were found. Patients with TNF-α-308 G/A_GG and IL-10-592 G/T_GT were at risk for F3/4. Lack of improvement of liver fibrosis was associated with lower baseline values of platelet count for genotypes TNF-α-308 G/A_GG and haplotype TT/GG of IL-10-1082 T/C and IL-10-592 G/T. Our study showed decreased liver fibrosis/inflammation and normalization of liver injury biomarkers after DAA treatment. It also points to the importance of suppressing the pro-inflammatory response by DAAs in the resolution of hepatitis C, contributing to the improvement of liver damage evaluated by transient elastography.

## 1. Introduction

The World Health Organization (WHO) estimates about 71 million (1.1% of the world population) people live with chronic hepatitis C infection.

Chronic hepatitis C (CHC) is a complex disease characterized by a clinical and pathological syndrome that has several causes and is accompanied by different degrees of hepatocellular necrosis and inflammation [1].

This disease is a significant cause of liver transplantation and liver-related death worldwide [2]. About 60% to 80% of people infected with the hepatitis C virus (HCV) develop a chronic stage of hepatitis C, with a 15% to 30% risk of developing liver cirrhosis or hepatocellular carcinoma (HCC) over 20 years [3]. HCC is known to be a leading cause of liver-related death and is the third most common cause of cancer death worldwide [4].

It is known that the outcome of CHC, namely liver fibrosis and its complications, is influenced by host factors (genetics, age, gender, obesity, steatosis, insulin resistance, diabetes, HVB, and HIV coinfections), viral factors (genotype and viral charge), and environmental factors (alcohol consumption, smoking, and physical activity) [4,5].

In the hepatic inflammatory process caused by HCV, several mechanisms are involved. Among them are host regulatory immune responses, which are mediated by cytokines that play an essential role against viral infection, and viral polypeptides, which interact with immune cells that are involved in the innate and adaptive response of infected individuals [4].

In CHC, the immune response changes over time and deteriorates as the disease progresses. Liver damage and chronic inflammation caused by HCV lead to the destruction of hepatocytes and a dominant pro-inflammatory and pro-fibrotic cytokine microenvironment. This fact, in turn, activates hepatic stellate cells, myofibroblasts, and fibroblasts, resulting in the deposition of extracellular matrix and fibrosis development [6].

HCV alters cytokine production, and determining these alterations may be essential to understand the mechanisms behind the resolution or persistence of the infection. However, several studies in this area had conflicting results as they reported a dominance of T helper 1 (Th1), T helper 2 (Th2), or both types of cytokines. The reason for this discrepancy may be related to HCV characteristics (genotype and virulence) and/or to host factors (duration of illness, genetic and immune background, and stage of liver disease regarding hepatic fibrosis and inflammation) [7,8].

Referring to the antiviral mechanism, tumor necrosis factor-alpha (TNF-α) is known to be associated with the enhancement of lymphocyte proliferation and with the stimulation of a targeted cytotoxic T lymphocyte (CTL) response against HCV within the liver [9].

The immune response associated with a Th2 cytokine profile is humoral and can inhibit antiviral effector mechanisms having an essential role in the progression and/or persistence of infection. In addition, Th2 cytokines generally impede the development of Th1 activity after acute viral infection, and they play a relevant role in host protection against tissue damage induced by Th1 cells [9].

A study by Lucey et al. suggested the involvement of a Th1-to-Th2 cytokine switch in the pathogenesis and persistence of virus infections [10].

Other studies reported an enhanced Th2 response in CHC as they revealed higher levels of Th2 cytokines that may be due to a systemic response or may be a result of local increased liver production and secretion to blood [11,12,13].

Regarding the immunopathogenesis of hepatitis C infection, the imbalance in the production of pro-inflammatory Th1 and anti-inflammatory Th2 cytokines may play a relevant role. Furthermore, after antiviral therapy, it can assess chronic liver disease development, progression, and outcome.

Despite a robust Th1 response in HCV-infected patients and mostly because of HCV’s high mutation rate, the infection persists and progresses into chronicity in most cases. In this situation, the host immune response is balanced in favor of a Th1 cytokine profile. As a result, it continues producing interleukine-2 (IL-2), interferon-gamma (IFN-γ), and TNF-α, which makes the host’s viral defense more aggressive but also causes chronic inflammation, necrosis, and liver damage [9].

Regarding liver disease progression, a study from 2001 demonstrated a shift to a predominant Th1 cytokine profile in patients with CHC as the liver damage progresses [14].

Interindividual differences in the pro-inflammatory/regulatory balance of cytokines appear to be dependent on allelic polymorphisms in the regulatory regions of cytokine genes [15].

It is believed that a vigorous pro-inflammatory response plays a crucial role in the resolution of hepatitis C. Still, this strong response can contribute to the appearance of liver damage. A balanced pro-inflammatory/regulatory cytokine response appears to play a key role in preserving host liver tissue [15].

### HCV Elimination with Direct-Acting Antiviral Therapy

The actual gold standard in treating HCV infection implies using direct-acting antivirals (DAAs). They act directly on the virus’s replicative cycle, inhibiting non-structural proteins associated with viral replication.

Recommended treatment regimens for adults are 8 to 12 weeks. HCV-RNA and aminotransferase testing are recommended at 12 or more weeks after completion of DAA therapy to assess treatment response. Undetectable HCV-RNA represents a sustained virologic response (SVR) and virologic cure. An SVR is generally associated with the normalization of liver enzymes, improvement or regression of liver necroinflammation and fibrosis, and improvement in liver function. Second-generation direct-acting antivirals (DAAs) have revolutionized the treatment of chronic HCV infection. DAA regimens show cure rates of more than 95%, minimal side effects, and shortened courses of therapy [16,17].

The primary objective of the study was to determine if HCV clearance with DAAs changes the severity of liver disease (fibrosis stage) evaluated by transient elastography (FibroScan), biochemical and cellular parameters of liver injury, and inflammatory/immune response (cytokines).

The secondary objectives were: (a) To evaluate the association of liver fibrosis with those biochemical and cellular parameters, cytokine plasma levels, and cytokine genetic polymorphisms before and after treatment; and (b) to identify at baseline which biochemical and cellular parameters, cytokines, and their functional genetic polymorphisms could predict the improvement of liver fibrosis after DAA treatment.

## 2. Results

### 2.1. Association of Biochemical and Cellular Parameters and Cytokines with Fibrosis Stage before and after DAA Treatment

Baseline biochemical and cellular parameters and cytokines were compared between patients with higher fibrosis stages (F3/4) and those with lower ones (F1/2) before DAA treatment. Patients with severe fibrosis showed higher values of alkaline phosphatase (ALP), aspartate aminotransferase (AST), alanine aminotransferase (ALT), gamma glutamyl transferase (γGT), and TNF-α as well as a low platelet count (Table 1).

Baseline biochemical and cellular parameters and cytokines were also compared after DAA treatment between patients with higher fibrosis stages (F3 and F4; F3/4) and those with lower ones (F1 and F2; F1/2). Patients with severe fibrosis showed increased ALP, AST, ALT, and γGT, as well as decreased platelet count values. However, cytokines showed no significant results (Table 2).

Biochemical and cellular parameters after DAA treatment were compared between patients with higher fibrosis stages (F3 and F4) and those with lower ones (F1 and F2). Patients with severe fibrosis showed increased values of ALP, AST, ALT, and γGT, as well as decreased values of platelet count (Table 3).

### 2.2. Fibrosis Stage before and after Treatment and Comparison of Biochemical and Cellular Parameters

Comparing the fibrosis stage before and after DAA treatment, we verify an improvement in liver fibrosis after DAA treatment. Before DAA treatment, patients showed a 2.410× risk for higher fibrosis stages (F3/4) (Table 4).

Comparing biochemical and cellular parameters before and after DAA treatment, patients showed lower values of ALP, AST, ALT, γGT, and TNFα after treatment, but the platelet counts remained equal (Table 5).

### 2.3. Association of Baseline Biochemical and Cellular Parameters and Cytokines with the Improvement of Fibrosis Stage after DAA Treatment

Within DAA-treated patients, 18.8% showed an improvement in liver fibrosis as they passed from higher fibrosis stages (F3 or F4) to lower fibrosis stages (F1 or F2). On the other hand, 79.0% maintained their initial fibrosis stage (58.9% F1 or F2 and 20.1% F3 or F4). Only 2.2% showed a worsening of fibrosis stage (F1/2 to F3/4).

Comparing baseline biochemical and cellular parameters of those who improved liver fibrosis with those who did not, we found increased values of platelet count for the first ones (Table 6).

Regarding only patients with cirrhosis (F4), 51.3% regressed in their fibrosis stage to mild, moderate, or severe (less than F4), while 48.7% stayed cirrhotic.

Comparing baseline biochemical parameters between these two groups and applying the same statistical tests as before (Table 6), we obtained the same results. Only the platelet counts showed increased values for those whose liver fibrosis improved compared with those who stayed cirrhotic.

### 2.4. Association of Cytokine Genetic Polymorphisms with Fibrosis Stage before and after DAA Treatment

Cytokine genetic polymorphisms were compared between patients with higher fibrosis stages (F3/4) and those with lower ones (F1/2) before and after DAA treatment.

Before treatment, patients with the CT or TT genotypes of TGF-β-509 C/T have protection for higher fibrosis stages. On the other hand, those homozygous TT for IL-10-1082 T/C and heterozygous GT for IL-10-592 G/T have 2.013× and 1.875× risk, respectively, of presenting severe fibrosis stages (F3,4).

After DAA treatment, patients heterozygous GA for TNF-α-308 G/A have a lower fibrosis stage (F1/2). Conversely, patients heterozygous GT for IL-10-592 G/T have a 3.043× risk of presenting higher stages of fibrosis (F3/4) after treatment than those with the GG genotype (Table 7).

### 2.5. Association of Cytokine Genetic Polymorphisms with the Improvement of Fibrosis Stage after DAA Treatment

Cytokine genetic polymorphisms were compared between patients who improved liver fibrosis (F3/4 to F1/2) with those who did not (maintained F3/4).

The frequency of patients with the GA genotype of TNF-α-308 G/A was higher in those who improved fibrosis stages (from F3/4 to F1/F2). This genotype was associated with a good prognosis regarding liver fibrosis after DAA treatment.

The same statistic regarding haplotypes of each gene and its association with the improvement of fibrosis stage (from F3/4 to F1/F2) after DAA treatment was made. We obtained significant results for the combination of the two polymorphisms of IL-10. The frequency of patients with the TT genotype for IL-10-1082 T/C and the GG genotype for IL-10-592 G/T was higher for those whose liver fibrosis improved. This haplotype was associated with a good prognosis regarding liver fibrosis after DAA treatment (Table 8).

We did the same comparing patients with regression of liver fibrosis F4 (cirrhosis) to F1, 2, or 3) with those who stayed cirrhotic (F4), and we obtained the same results.

## 3. Discussion

Responding to the primary objective, HCV clearance with DAAs changes the severity of liver disease (fibrosis stage), as we found an increase in F1/2 patients after DAA treatment. In addition, it also changes the biochemical and cellular parameters of liver injury and the inflammatory/immune response (cytokines), as we found a normalization of transaminase and platelet count values and a decrease in TNF-α plasma concentration.

Regarding the secondary objectives, we found that higher fibrosis stages (F3/4), before and after DAA treatment, were associated with higher values of transaminases and lower values of platelet count. TNF-α plasma concentration was only increased in F3/4 patients before DAA treatment.

TGF-β and IL-10 genetic polymorphisms were associated with higher fibrosis stages before DAA treatment, and TNF-α and IL-10 genetic polymorphisms showed the same association after DAA treatment.

The improvement of liver fibrosis after DAA treatment was associated with higher baseline values of platelet count as well as TNF-α and IL-10 genetic polymorphisms.

Liver disease caused by HCV infection translates into structural changes in the liver that result in several stages of liver fibrosis and serum non-invasive biomarkers of liver damage, necroinflammation, and fibrosis [18]. As expected, in our study, higher values of AST, ALT, and ALP were associated with more advanced fibrosis stages before and after DAA treatment. These results agree with other studies that report a normalization of these biomarkers after HCV elimination [19,20].

Another important enzyme is γGT. Eminler et al. stated that γGT values are increased in higher fibrosis stages [21]. In our sample, higher values of γGT were also associated with more advanced fibrosis stages before and after DAA treatment, as also showed by Everhart et al. [22].

Platelets play a fundamental role in hemostasis, found at the beginning of the coagulation cascade in response to vascular injury. When liver damage occurs, platelets are actively recruited to the liver and will play a vital role in tissue regeneration [23]. Thrombocytopenia in individuals with chronic advanced liver disease is caused by the accumulation of platelets in the spleen due to portal hypertension, reduced production of thrombopoietin by the injured liver, and loss of hematopoietic function in bone marrow due to alcohol abuse or viral infection [24,25].

A higher baseline number of platelets was associated with improved liver fibrosis, emphasizing the importance of this blood component in liver regeneration after HCV clearance.

Cytokines are essential in controlling the body’s immune response to pathogens. HCV alters the production of anti- and pro-inflammatory cytokines, and the imbalance in the production of pro-inflammatory Th1 and anti-inflammatory Th2 cytokines can be used for assessing chronic liver disease development, progression, and outcome [10]. TNF-α is a potent immunomodulator and an inflammatory cytokine produced during acute phase inflammation. It plays a significant role in mediating inflammatory conditions in the liver. Several studies have observed that individuals with the hepatitis C virus and hepatocellular carcinoma have higher levels of TNF-α, supporting the relevance of this pro-inflammatory cytokine in hepatocarcinogenesis and HCV infection. High levels of TNF-α have been associated with increased inflammatory activity and more severe illness in individuals with chronic hepatitis C. It has also been shown that TNF-α activates hepatic stellate cells and can modify the phenotype of activated myofibroblasts, thus causing an accumulation of molecules in the extracellular matrix, leading to fibrogenesis and fibrosis progression [26].

A study by Chen et al. found high levels of this cytokine in individuals with chronic hepatitis C [27]. Moreover, Crespo et al. reported intrahepatic mRNA for TNF-α and other cytokines expressed in excess. The level of expression correlated with higher stages of fibrosis [28]. In agreement with previous studies, our research showed an association between increased values of this cytokine and higher fibrosis stages evaluated by transient elastography before DAA treatment, indicating the importance of improving the inflammatory process in reducing fibrosis staging. It also revealed a decrease in TNF-α after HCV clearance, indicating a reduction in the inflammatory process, as shown in Table 5.

Transforming growth factor-beta (TGF-β) is a cytokine secreted by hepatic stellate cells, fibroblasts, Kupffer cells, and M2 macrophages that regulate cell growth, differentiation, and proliferation [29,30].

This cytokine is multifunctional, showing pro-fibrotic, anti-inflammatory, and immunosuppressive effects. The balance of these effects is essential for homeostasis in the tissues where this cytokine is present. Therefore, when there is an aberrant expression of TGF-β, it will trigger the development of fibrogenesis and fibrosis progression. On the other hand, a deficiency of this cytokine can also cause the development of diseases [31]. Furthermore, this cytokine plays a significant role in hepatic fibrogenesis, as it will act as a potent inducer of extracellular matrix accumulation [32].

TGF-β is a central regulator in chronic liver disease and is involved in fibrogenesis. Levels of this cytokine induced by liver damage and platelet activation mediate the activation of hepatic stellate cells and fibroblasts with the production of myofibroblasts and the deposition of extracellular matrix [33]. Although our study revealed an improvement in liver fibrosis after DAA treatment, this was not followed by a decrease in the amount of TGF-β. As we evaluated patients after just six months of therapy, we will likely be unable to see changes in the amount of this cytokine. This is because the regression of inflammation comes before to the regression of liver fibrosis, which may induce a decrease in liver stiffness presented by elastography values [34,35].

Interleukine-10 (IL-10) is an immunoregulatory cytokine produced mainly by monocytes, macrophages, and T cells [36]. It is a Th2 anti-inflammatory cytokine, which plays a crucial role in regulating immune responses. It influences the balance of Th1/Th2 cells, negatively controlling the response of Th1 lymphocytes and suppressing the action of pro-inflammatory cytokines [37,38].

IL-10 is a multifunctional cytokine and a potent regulator of immune function through its anti-inflammatory and anti-fibrotic action. IL-10 is believed to be one of the crucial cytokines mediating the host’s immune response to HCV infection [39].

The deficiency or the overproduction of IL-10 can lead to several pathological conditions. For example, a low level can increase inflammatory reactions and help spontaneous HCV clearance. On the other hand, its overexpression can inhibit immune response, enabling viral evasion and increasing susceptibility to reinfections [37,40].

Some studies have demonstrated that IL-10 is involved in the lack of HCV eradication and, consequently, in the progression to chronicity. In addition, this cytokine plays an essential role in suppressing immune response in the early phases of HCV infection, as it suppresses HCV-specific effectors cluster of differentiation 4 (CD4 +) and cluster of differentiation 8 (CD8 +) T cells [41,42,43].

Regarding liver disease progression and despite sparse evidence, IL-10 seems to protect against fibrosis. A study from 2013 reported an inverse correlation between IL-10 levels and fibrosis stages in patients with CHC, while another showed that treatments with an IL-10 agonist reveal a decrease in fibrosis progression in chronic hepatitis C virus infection [26,44].

IL-10 was significantly higher in patients with chronic hepatitis (B, C, or both) than in healthy controls [45].

Two studies from 1996 and 2000 showed a higher expression of IL-10 in patients with milder forms of hepatitis C than those with severe liver disease and a correlation between the downregulation of IL-10 and CHC [45,46]. In addition, IL-10 can decrease T cells’ expression of pro-inflammatory cytokines (IL-2, IFN-γ, and TNF-α) and downregulate the expression of collagen 1, exerting its anti-fibrotic effects [15].

Cytokine levels may be modulated by genetic polymorphisms within their encoded genes.

A single nucleotide polymorphism at the −308 position within the TNF-α promoter is characterized by the presence of two alleles, G and A, with allele G having the highest frequency in populations and the AA genotype having the highest expression of TNF-α [47,48,49]. This polymorphism is associated with severe inflammatory states, autoimmune diseases, infectious diseases, and malignant tumors. In addition, some studies have shown that the AA genotype significantly correlates with the increased risk for cirrhosis and hepatocellular carcinoma [4,50,51].

Anther polymorphism within the TNF-α promoter, at −238 position, is characterized by the presence of two alleles, G and A, with allele G having the highest frequency and with the AA genotype having the highest expression of TNF-α [52,53]. It was demonstrated that individuals with the GG genotype had significantly higher liver stiffness and liver cirrhosis than individuals with GA or AA genotypes [54]. However, Moreira et al. and Yee et al. reported a protective role for high fibrosis stages in homozygous GG and an association between this specific polymorphism and higher fibrosis stages [55,56].

Regarding the TGF-β gene, some single nucleotide polymorphisms that affect cytokine expression/level were also identified. One of them is a single nucleotide polymorphism characterized by a C to T switch at −509 position within the TGF-β promoter, with allele C having the highest frequency and the TT genotype having the highest expression of TGF-β [57,58]. Some studies revealed that the CT and TT genotypes are more prevalent in individuals with high fibrosis stages and with hepatocellular cancer. It has also been reported that homozygosity TT is associated with susceptibility to chronic hepatitis virus infection [28,59].

Another functional polymorphism within the TGF-β promoter is located at position 29. It consists of an exchange of T to C, with allele T having the highest frequency and the CC genotype having the highest expression of TGF-β [57,60].

Several studies reported an association between allele T of TGF-β -509 C/T and higher fibrosis stages with an increase in cytokine expression [61,62,63]. Our research found a statistically significant association between the CC genotype and higher fibrosis stages, as shown in Table 7. However, it is known that the reduced expression of TGF-β may also be associated with fibrotic and autoimmune liver diseases, thus explaining the association obtained in our study [30].

We have also identified some functional single nucleotide polymorphisms within the IL-10 gene. One of them is located at the −1082 position within the IL-10 promoter and is characterized by the presence of alleles T and C, with allele T having the highest frequency and genotype CC the highest expression [36,64,65]. Regarding the susceptibility to chronic hepatitis C, some studies reveal that the TT genotype was frequently observed in individuals with this pathology. Moreover, this genotype was more frequent in individuals with hepatocellular carcinoma [4,37,66,67]. On the other hand, the CC genotype being associated with an increased risk for HCV infection may be due to a greater expression of IL-10 facilitating viral evasion [65,66].

Another single nucleotide polymorphism described within the IL-10 promoter is found at the −592 position. It consists of a T to G exchange, with allele G having the highest frequency and genotype GG having the highest expression of IL-10 [66,67]. Scientific evidence shows that homozygous TT individuals are more prone to developing more severe forms of chronic liver disease and hepatocellular cancer [4]. In addition, the GG genotype was found to have a more significant association with milder liver inflammatory processes [36].

It was found in the article by Knapp et al. that allele C of IL-10-1082 T/C increased the expression of this cytokine [68]. On the other hand, regarding IL10-592 G/T, Sun et al. showed that allele T decreased the expression of IL-10 [66].

Our study revealed an association between the CC genotype of IL-10-1082 T/C and the GT genotype of IL10-592 G/T and higher fibrosis stages. The results for the first polymorphism were also observed in the studies carried out by Liu et al. and da Silva et al. [69,70]. For the second one, Gao et al. also found that the GT genotype increases the risk for higher fibrosis and has a significant synergistic effect on the lesion inflammatory activity in HCV-infected individuals [71].

Regarding the association between studied polymorphisms and serum enzymes of liver damage, our study revealed an association between the CC or CT genotypes of TGF-β 29 T/C and higher values of γGT and ALP. It was also found that individuals with these genotypes were at 1.8× risk of having γGT levels above the reference value compared to individuals with the TT genotype. Many patients with chronic HCV infection had elevated serum GGT levels. This enzyme seemed helpful as an indirect marker of more advanced liver disease in chronic hepatitis C [72].

Studies point to the relevant role of this cytokine in the various stages of the progression of chronic liver disease, from initial liver damage to inflammation, fibrosis, cirrhosis, and hepatocellular carcinoma [33]. Therefore, allele C is associated with an increased expression of TGF-β. An association between this specific allele of TGF-β 29 T/C and increased levels of γGT and ALP was expected, as they indicate more significant liver damage associated with higher fibrosis stages.

## 4. Materials and Methods

A group of 329 patients with chronic hepatitis C infection were prospectively studied.

Characterization of chronic hepatitis C patients before and after DAA treatment is described in Table 9.

The subjects in the study were selected, examined, adequately informed, and provided consent following the WMA Helsinki Declaration [73].

Inclusion criteria (before treatment): positive RNA and anti-HCV for more than 6 months; age 18 years or older; and informed consent.

Inclusion criteria (after treatment): sustained response (HCV-RNA undetectable 3 and 6 months after viral load 0 UI/mL according to European Association for the Study of Liver (EASL) guidelines).

Exclusion criteria: other chronic liver diseases (viral hepatitis A and/or B, autoimmune diseases, and other genetic and/or metabolic diseases); concurrent infection with HIV; alcohol consumption >40 gr/day; pregnant women; and individuals with impaired intellectual capacity.

### 4.1. Liver Fibrosis Evaluation

The liver fibrosis stage was evaluated by transient elastography (TE) using a FibroScan^®^ device (Echosens, Paris, France) with a 5-MHz ultrasound transducer mounted on the axis of a vibrator. The median value of 10 successful acquisitions was expressed in kilopascals (kPa), with a success rate of at least 60% and an interquartile range (IQR) lower than 30%. Cut-off values were validated in the Gastroenterology and Hepatology Department, Hospital de Santa Maria, Lisbon, Portugal (analysis of 110 patients, Scheuer classification): 5.43 kPa for F ≥ 2 (PPV 0.78; NPV 0.67); 8.18 kPa for F ≥ 3 (PPV 0.95 NPV 0.93); 12 kPa for F = 4 (PPV 0.93; NPV 0.93) [74].

### 4.2. Biochemical Evaluation

Serum biochemical parameters were evaluated before and after antiviral treatment using standard methods from the hospital laboratory department (reference values described): alkaline phosphatase (AP ≤ 129 UI/L), aspartate aminotransferase (AST ≤ 34 IU/L), alanine aminotransferase (ALT ≤ 49 IU/L), γ-glutamyl-transpeptidase (γGT < 38 IU/L), and platelet count (≥150,000/µL).

Serum HCV-RNA was evaluated by Real-Time PCR Taqman from Roche Diagnostics (test sensitivity <15 IU/mL) and genotypes by Hybridization Probes—LiPA “Line Probe Assay”; VERSANT™ HCV LiPA 2.0; 10313066 (Siemens Healthcare Diagnostics); software 10291328; Erlangen, Germany.

### 4.3. Cytokine Evaluation

Plasma concentrations of TNF-α, TGF-β, and IL-10 cytokines before and after antiviral treatment were measured by ELISA (enzyme-linked immunoabsorbent assay) with Quantikine^®^ high sensitivity kits from R&D Systems; Arium, Portugal (Human TNF-α immunoassay Catalog Number HSTE00D, Sensitivity: 0.049 pg/mL; Assay range: 0.2–10 pg/mL; Human TGF-β immunoassay Catalog Number DB100C, Sensitivity: 5.5 pg/mL; Assay range: 31.3–2000 pg/mL; Human IL-10 immunoassay Catalog Number D1000B, Sensitivity: 0.17 pg/mL; Assay range: 0.8–50 pg/mL).

### 4.4. DNA Extraction

Blood was collected in EDTA and stored at −20 °C until analysis. DNA was isolated from leukocytes by an adapted non-enzymatic DNA extraction procedure [75].

### 4.5. Genetic Polymorphism Identification

TGF-β-509 C/T (rs1800469) and TGF-β 29 T/C (rs1800470) genetic polymorphisms were determined by PCR-RFLP (polymerase chain reaction—restriction fragment length polymorphism). The remaining polymorphisms, TNF-α-308 G/A (rs1800629), TNF-α-238 G/A (rs361525), IL-10-1082 T/C (rs1800896), and IL-10-592 G/T (rs1800872), were determined by endpoint genotyping in a LightCycler 480II (Roche Diagnostics). All the reagents and genotyping conditions are described in Table 10, Table 11, Table 12 and Table 13.

### 4.6. Statistical Analysis

Statistical analysis was performed by SPSS 24.0 for Windows. Data were inserted into a database built in this same program, safeguarding the confidentiality of the participants’ identities.

Two groups were established for the analysis of the liver fibrosis stage: patients with mild and moderate fibrosis (F1 and F2) and patients with severe fibrosis and cirrhosis (F3 and F4).

Regarding the studied genetic polymorphisms, they were statistically evaluated as three groups (codominant model), and we grouped the heterozygous genotype with the dominant genotype (dominant model) and with the recessive genotype (recessive model). In addition, for polymorphisms within the same gene, haplotype analysis was performed.

Descriptive analysis was performed assuming univariate and bivariate analysis. For continuous variables, normality was first tested by the non-parametric Kolmogorov–Smirnov test. Then, as all variables showed normal distribution, they were analyzed using parametric tests and were described using means and 95% confidence intervals (95% CI). To describe the categorical variables, absolute and relative frequencies were used. In the bivariate analysis, categorical variables were compared using the Chi-square test or Fisher’s exact test. The Odds Ratio (OR) was calculated with their respective confidence intervals whenever justifiable. For continuous variables, statistical comparisons were performed using the non-parametric Mann–Whitney and Kruskal–Wallis tests. To make comparisons before and after DAA treatment, we used a paired sample test. It was considered a statistically significant result for a *p*-value < 0.05.

## 5. Conclusions

There was an association between the studied cytokine gene polymorphisms and the fibrosis stage before and after HCV clearance.

Before treatment, patients with the CC genotype of TGF-β-509 C/T, CC genotype of IL-10-1082 T/C, and GT genotype of IL-10-592 G/T were at risk for higher fibrosis stages.

After treatment, patients with the GG genotype of TNF-α-308 G/A and GT genotype of IL-10-592 G/T were at risk for higher fibrosis stages.

The improvement of liver fibrosis after treatment was associated with the presence of allele A of TNF-α-308 G/A and non-improvement with haplotype TT/GG of IL-10-592 G/T and IL-10-1082 T/C.

This is the first study regarding the association of cytokine gene polymorphisms and the improvement of liver fibrosis after DAA treatment.

Our study also showed a decrease in liver fibrosis/inflammation and a normalization of liver injury biomarkers after DAA treatment. However, TNF-α improved at six months of follow-up but TGF-β did not. These results may indicate not only a non-effective regression of fibrosis but also an improvement of liver inflammation associated with decreased elastography values.

It points to the importance of suppressing the pro-inflammatory response by DAAs in the resolution of hepatitis C, contributing to the improvement of liver damage evaluated by transient elastography.

The results of this study emphasize the importance of following the patients with high values of elastography before treatment, even after the elimination of HCV and low values of elastography. They may be at risk for liver complications, namely hepatocellular carcinoma, as these low values may not yet reflect a reduction in liver fibrosis but in liver inflammation.

## Figures and Tables

**Table 1:** Association of baseline biochemical and cellular parameters and cytokines (continuous variables) with fibrosis stage before DAA treatment.

Parameter	F_1/2_		F_3/4_		*p*-Value *
	Mean	95% CI	Mean	95% CI	
ALP (UI/l)	72.06	[68.60–75.51]	93.15	[85.03–101.28]	<0.001
AST (UI/l)	49.67	[45.48–53.86]	88.26	[77.27–99.26]	<0.001
ALT (UI/l)	79.91	[70.34–89.48]	116.07	[98.79–133.36]	<0.001
γGT (UI/l)	55.26	[48.86–61.66]	118.53	[95.74–141.33]	<0.001
Platelet count (U/µL)	2.22 × 10^5^	[2.13 × 10^5^–2.31 × 10^5^]	1.75 × 10^5^	[1.61 × 10^5^–1.90 × 10^5^]	<0.001
IL-10 (pg/mL)	4.55	[1.59–7.50]	2.11	[1.46–2.76]	0.199
TNF-α (pg/mL)	2.84	[1.85–3.82]	3.13	[2.17–4.08]	0.034
TGF-β (pg/mL)	1475.43	[1335.57–1615.29]	1306.63	[1131.29–1481.97]	0.169

*—Mann–Whitney Test; 95% CI—95% confidence interval for mean.

**Table 2:** Association of baseline biochemical and cellular parameters and cytokines (continuous variables) with fibrosis stage after DAA treatment.

Parameter	F_1/2 (After DAA Treatment)_		F_3/4 (After DAA Treatment)_		*p*-Value *
	Mean	95% CI	Mean	95% CI	
ALP (UI/l)	74.74	[69.95–79.52]	100.17	[85.03–101.28]	0.001
AST (UI/l)	50.84	[44.29–57.40]	81.23	[66.65–95.81]	<0.001
ALT (UI/l)	70.48	[60.03–80.92]	95.03	[72.80–117.26]	0.003
γGT (UI/l)	77.24	[61.40–93.08]	128.97	[87.63–170.36]	0.002
Platelet count (U/µL)	2.18 × 10^5^	[2.06 × 10^5^–2.30 × 10^5^]	1.60 × 10^5^	[1.27 × 10^5^–1.92 × 10^5^]	<0.001
IL-10 (pg/mL)	3.90	[1.63–6.18]	1.92	[1.00–2.83]	0.394
TNF-α (pg/mL)	2.46	[1.53–3.39]	1.92	[1.45–2.38]	0.323
TGF-β (pg/mL)	1602.66	[1408.73–1796.59]	1534.26	[1163.77–1904.74]	0.732

*—Mann–Whitney Test; 95% CI—95% confidence interval for mean.

**Table 3:** Association of biochemical and cellular parameters after DAA treatment (continuous variables) with fibrosis stage after DAA treatment.

Parameter _(After DAA Treatment)_	F_1/2 (After DAA Treatment)_		F_3/4 (After DAA Treatment)_		*p*-Value *
	Mean	95% CI	Mean	95% CI	
ALP (UI/l)	69.08	[64.76–73.39]	81.76	[70.49–93.03]	0.020
AST (UI/l)	23.02	[21.56–24.48]	30.13	[22.42–37.85]	0.002
ALT (UI/l)	21.73	[19.57–23.89]	29.37	[20.01–38.72]	0.009
γGT (UI/l)	21.52	[18.90–24.15]	62.55	[31.26–93.84]	<0.001
Platelet count (U/µL)	2.14 × 10^5^	[1.99 × 10^5^–2.30 × 10^5^]	1.67 × 10^5^	[1.28 × 10^5^–2.04 × 10^5^]	<0.001
IL-10 (pg/mL)	3.27	[0.68–5.86]	2.41	[1.15–3.67]	0.430
TNF-α (pg/mL)	1.60	[1.26–1.94]	1.44	[0.92–1.97]	0.979
TGF-β (pg/mL)	1370.99	[1156.11–1585.87]	1394.19	[951.76–1836.61]	0.714

*—Mann–Whitney Test; 95% CI—95% confidence interval for the mean.

**Table 4:** Fibrosis stage before and after DAA treatment.

	Fibrosis Stage				
	F1/2	F3/4	*p*-Value *	OR	95% CI
Before DAA treatment; n (%)	82 (61.2)	52 (38.8)	0.001	2.410	[1.416–4.100]
After DAA treatment; n (%)	114 (79.2)	30 (20.8)		1	

*—Fisher exact test; OR—odds ratio; 95% CI—95% confidence interval.

**Table 5:** Biochemical and cellular parameters before and after DAA treatment (continuous variables).

Parameter	Before DAA Treatment		After DAA Treatment		*p*-Value *
	Mean	95% CI	Mean	95% CI	
ALP (UI/l)	80.37	[74.65–86.10]	71.70	[67.48–75.92]	<0.001
AST (UI/l)	57.70	[51.36–64.04]	24.59	[22.52–26.67]	<0.001
ALT (UI/l)	76.02	[66.48–85.55]	23.38	[20.70–26.06]	<0.001
γGT (UI/l)	88.27	[72.68–103.85]	30.62	[23.10–38.14]	<0.001
Platelet count (U/µL)	2.05 × 10^5^	[1.92 × 10^5^–2.17 × 10^5^]	2.04 × 10^5^	[1.89 × 10^5^–2.18 × 10^5^]	0.456
IL-10 (pg/mL)	3.68	[1.61–5.74]	3.49	[0.91–6.06]	0.796
TNF-α (pg/mL)	2.08	[1.53–2.63]	1.59	[1.29–1.88]	0.035
TGF-β (pg/mL)	1655.82	[1448.98–1862.66]	1419.52	[1219.83–1619.20]	0.280

*—Mann–Whitney Test; 95% CI—95% confidence interval for mean.

**Table 6:** Comparison of baseline biochemical and cellular parameters and cytokines (continuous variables) between patients whose liver fibrosis improved and those whose did not.

Baseline Parameter	F_3/4 (Before DAA Treatment)_ to F_1/2 (After DAA Treatment)_		F_3/4 (Before DAA Treatment)_ to F_3/4 (After DAA Treatment)_		*p*-Value *
	Mean	95% CI	Mean	95% CI	
ALP (UI/l)	83.04	[71.10–94.98]	101.30	[81.13–121.47]	0.179
AST (UI/l)	79.96	[57.10–102.81]	84.85	[69.36–100.34]	0.234
ALT (UI/l)	101.79	[70.65–132.94]	99.56	[75.48–123.63]	0.692
γGT (UI/l)	134.63	[78.44–190.81]	126.37	[82.01–170.73]	0.727
Platelet count (U/µL)	2.11 × 10^5^	[1.78 × 10^5^–2.44 × 10^5^]	1.49 × 10^5^	[1.16 × 10^5^–1.82 × 10^5^]	0.003
IL-10 (pg/mL)	2.26	[1.17–3.36]	1.92	[1.00–2.83]	0.606
TNF-α (pg/mL)	2.42	[0.98–3.86]	1.93	[1.44–2.42]	0.608
TGF-β (pg/mL)	1346.71	[1031.86–1661.56]	1499.27	[1106.72–1891.83]	0.595

*—Mann–Whitney Test; 95% CI—95% confidence interval for mean.

**Table 7:** Association of cytokine genetic polymorphisms with fibrosis stage before and after DAA treatment.

Polymorphism	Genotype	F1/2 _(Before DAA Treatment)_		F3/4 _(Before DAA Treatment)_		*p*-Value	OR	CI 95%	F1/2 _(After DAA Treatment)_		F3/4 _(After DAA Treatment)_		*p*-Value	OR	CI 95%
		N	%	N	%				N	%	N	%			
TNF-α-308 G/A	GG	129	68.25	64	66.67	0.863 **	-	-	66	66.00	25	86.21	0.039 *	1	-
	GA	56	29.63	29	30.21				34	34.00	4	13.79		0.311	[0.100–0.965]
	AA	4	2.12	3	3.13										
TNF-α-238 G/A	GG	157	91.81	78	87.64	0.691 *	-	-	86	91.81	23	87.64	1.000 *	-	
	GA	14	8.19	11	12.36				11	8.19	3	12.36			
TGF-β-509 C/T	CC	62	31.16	46	45.10	0.018 **	NA	-	34	31.16	13	45.10	0.274 **	-	
	CT	103	51.76	48	47.06				51	51.76	9	47.06			
	TT	34	17.09	8	7.84				14	17.09	4	7.84			
	CC	62	31.16	46	45.10	0.022 *	1	-							
	CT or TT	137	68.84	56	54.90		0.551	[0.337–0.901]							
TGF-β 29 T/C	TT	65	34.76	35	37.23	0.473 **	-	-	34	34.76	11	37.23	0.797 **	-	
	CT	92	49.20	49	52.13				47	49.20	11	52.13			
	CC	30	16.04	10	10.64				15	16.04	4	10.64			
IL-10-1082 T/C	TT	59	34.50	31	32.29	0.099 **	-	-	38	34.50	6	32.29	0.170 **	-	
	TC	90	52.63	43	44.79				45	52.63	17	44.79			
	CC	22	12.87	22	22.92				16	12.87	7	22.92			
	TT or TC	149	87.13	74	77.08	0.040 *	1	-							
	CC	22	12.87	22	22.92		2.013	[1.048–3.870]							
IL-10-592 G/T	GG	85	50.30	34	35.05	0.021 *	1	-	52	50.30	8	35.05	0.021 *	1	-
	GT	84	49.70	63	64.95		1.875	[1.121–3.137]	47	49.70	22	64.95		3.043	[1.237–7.485]

*—Fisher exact test; **—Pearson chi-square test; For dominant and recessive models, only significant results are shown; NA—not applicable; OR—odds ratio; CI 95—95% confidence interval.

**Table 8:** Association of cytokine genetic polymorphisms and haplotypes with the improvement of fibrosis stage after DAA treatment.

Polymorphism	Genotype	F_3/4 (Before DAA Treatment)_ to F_1/2 (After DAA Treatment)_		F_3/4 (Before DAA Treatment)_ to F_3/4 (After DAA Treatment)_		*p*-Value	OR	CI 95%
		N	%	N	%			
TNF-α-308 G/A	GG	10	45.45	23	88.46	0.002 *	1	-
	GA	12	54.55	3	11.54		0.109	[0.025–0.471]
TNF-α-238 G/A	GG	16	39.02	22	91.67	0.439 *	-	-
	GA	25	60.98	3	8.33			
TGF-β-509 C/T	CC	11	45.83	12	52.17	0.208 **	-	-
	CT	12	50.00	7	30.43			
	TT	1	4.17	4	17.40			
TGF-β 29 T/C	TT	10	43.48	9	39.13	0.881 **	-	-
	CT	11	47.83	11	47.83			
	CC	2	8.69	3	13.04			
IL-10-1082 T/C	TT	10	40.00	5	18.52	0.050 **	-	-
	TC	8	32.00	16	59.26			
	CC	7	28.00	6	22.22			
IL-10-592 G/T	GG	11	47.83	8	29.63	0.247 *	-	-
	GT	12	52.17	19	70.37			
IL-10-1082 T/C + IL-10-592 G/T	TT/GG	8	34.78	2	7.41	0.030 *	1 6.667	- [1.247–35.647]
	Other	15	65.22	25	92.59			

*—Fisher exact test; **—Pearson chi-square test; OR—odds ratio; CI 95—95% confidence interval.

**Table 9:** Characterization of chronic hepatitis C patients before and after DAA treatment.

	Before DAA Treatment (n = 329)		After DAA Treatment (n = 134)	
Parameter	Mean	95% CI	Mean	95% CI
Age (years)	48.93	[47.57–50.28]	53.42	[51.47–55.36]
BMI (kg/m^2^	25.25	[24.80–25.06]	25.06	[20.78–26.10]
ALP (UI/l)	79.52	[75.73–83.31]	71.86	[67.66–76.06]
AST (UI/l)	63.06	[57.98–68.15]	24.61	[22.55–26.67]
ALT (UI/l)	92.53	[83.71–101.36]	23.44	[20.78–26.10]
γGT (UI/l)	77.48	[67.93–87.03]	30.54	[23.08–38.00]
Platelet count (U/µL)	2.05 × 10^5^	[1.97 × 10^5^–2.13 × 10^5^]	2.04 × 10^5^	[1.97 × 10^5^–2.18 × 10^5^]
**Parameter**	**n**	**%**	**n**	**%**
Gender				
Female	124	37.7	58	43.3
Male	205	62.3	76	56.7
HCV genotype				
1 and 4	257	78.2	117	87.3
2 and 3	72	21.8	17	12.7
Liver fibrosis				
F_1/2_	210	65.0	104	77.6
F_3/4_	115	35.0	30	22.4

95% CI—95% confidence interval for mean; n—number of patients; %—percentage of patients.

**Table 10:** Primers and amplification fragments for TGF-β polymorphisms.

Polymorphism	Primers	Amplification Fragment
TGF-β-509 C/T	Forward 5′—TGA TCC AGA TGC GCT GTG GCT T—3′ Reverse 5′—CTC AGT AAA GGA GAG CAA TTC T—3′	280 pb
TGF-β 29 T/C	Forward 5′—ACC ACA CCA GCC CTG TTC GCG C—3′ Reverse 5′—AGC CAC AGC AGC GGT AGC AGG A—3′	107 pb

**Table 11:** Amplification conditions and reagents for TGF-β polymorphisms.

Polymorphism	Amplification Conditions	Reagents
TGF-β-509 C/T	HotStart—94 °C, 2 min	200 ng DNA and H_2_O up to 10 µL
	35 cycles Denaturation: 94 °C, 45 s Annealing: 54 °C, 45 s Extension: 72 °C, 60 s	DNA: 10 µL, 200 ng Primer Forward (Invitrogen): 10 pmol Primer Reverse (Invitrogen): 10 pmol Green Taq PCR Master Mix (Thermofisher): 12.5 μL Deionized H_2_O: 0.5 μL
	1 cycle Final extension: 72 °C, 5 min	
TGF-β 29 T/C	HotStart—94°C, 2 min	200 ng DNA and H_2_O up to 10 µL
	35 cycles Denaturation: 94 °C, 45 s Annealing: 67 °C, 45 s Extension: 72 °C, 60 s	DNA: 10 µL, 200 ng Primer Forward (Invitrogen): 10 pmol Primer Reverse (Invitrogen): 10 pmol Green Taq PCR Master Mix (Thermofisher): 12.5 μL Deionized H_2_O: 0.5 μL
	1 cycle Final extension: 72 °C, 5 min	

**Table 12:** Restriction conditions, reagents, and restriction fragments/genotypes for TGF-β polymorphisms.

Polymorphism	Restriction Conditions	Reagents	Restriction Fragments/Genotypes
TGF-β-509 C/T	16 h; 37 °C	Amplification product: 10 μL Eco 81I (10 U/μL) (Fermentas): 10 U Buffer (10×) (Fermentas): 2 μL Deionized H_2_O: 7 μL	CT (280 pb + 226 pb + 54 pb) CC (226 pb + 54 pb) TT (280 pb)
TGF-β 29 T/C	16 h; 37 °C	Amplification product: 10 μL MbiI (10 U/μL) (Fermentas): 10 U Buffer (10×) (Fermentas): 2 μL Deionized H_2_O: 7 μL	CT (107 pb + 84 pb + 23 pb) CC (84 pb + 23 pb) TT (107 pb)

**Table 13:** Endpoint genotyping conditions and reagents for TNF-α and IL-10 polymorphisms.

Polymorphism	Endpoint Genotyping Conditions	Reagents
TNF-α-308 G/A TNF-α-238 G/A IL-10-1082 T/C IL-10-592 G/T	Pre-incubation: 95 °C, 10 min	DNA (5 ng): 6.75 µL TaqMan ™ SNP (Thermofisher): 0.75 µL TaqMan ™ Master Mix (Thermofisher): 7.5 μL
	40 cycles Amplification: 95 °C, 5 s; 60 °C, 1 min	

## Data Availability

The datasets used and or analyzed during the current study are available from the corresponding author upon reasonable request.
